# Patient-Reported Firearm Access Prior to Suicide Death

**DOI:** 10.1001/jamanetworkopen.2021.42204

**Published:** 2022-01-10

**Authors:** Julie E. Richards, Jennifer M. Boggs, Ali Rowhani-Rahbar, Elena Kuo, Marian E. Betz, Jennifer F. Bobb, Gregory E. Simon

**Affiliations:** 1 Kaiser Permanente Washington Heath Research Institute, Seattle, Washington; 2 Department of Health Services, University of Washington, Seattle; 3 Kaiser Permanente Colorado Institute for Health Research, Aurora; 4 Department of Epidemiology, School of Public Health, University of Washington, Seattle; 5 Firearm Injury and Policy Research Program, Harborview Injury Prevention & Research Center, Seattle, Washington; 6 Department of Emergency Medicine, University of Colorado School of Medicine, Aurora; 7 Psychiatry and Behavioral Sciences, University of Washington, Seattle; 8 Kaiser Permanente Washington Department of Mental Health & Wellness, Seattle, Washington

## Abstract

This case series examines how individuals who died by suicide responded to a survey question regarding access to firearms before death.

## Introduction

Firearms are the most common means of suicide death in the US.^1^ Major medical associations recommend health care providers counsel at-risk patients to limit firearm access.^2^ However, no national practice recommendations exist for implementing standardized firearm access screening.^3^ Health care systems more commonly rely on clinicians to ask patients about firearm access and ownership at their discretion.^3^ In 2015, Kaiser Permanente Washington added the question, “Do you have access to guns? (yes/no),” to a standard mental health (MH) monitoring questionnaire to support suicide risk identification and safety planning.^4^ Our objective was to evaluate whether and how suicide decedents who received ambulatory care answered the question about firearm access in the year prior to death.

## Methods

This population-based case series utilized Washington State death records and electronic health record data to identify Kaiser Permanente Washington patients who received outpatient care within the year prior to suicide death. Cause-of-death indicators for firearm vs other suicide means were defined using *International Statistical Classification of Diseases and Related Health Problems, Tenth Revision* codes for intentional self-harm by firearm; sociodemographic and clinical characteristics were extracted from electronic health data (eMethods in the Supplement). Between January 1, 2016, and December 31, 2019, the MH monitoring questionnaire was routinely used and recorded in the electronic health record for all MH specialty encounters, urgent care encounters per provider discretion, and primary care encounters following MH integration for all patients with an active MH or substance use disorder (SUD) diagnosis.^5^ We report the proportions of patients who (1) had MH or SUD diagnoses, (2) received the firearm question, (3) answered the question, and (4) reported access. We stratified findings by firearm and other suicide methods, because opportunities for suicide prevention may differ between these groups. The Kaiser Permanente institutional review board approved this study and waived the need for patient informed consent, because use of this protected health information involved no more than a minimal risk to the privacy of individuals. This study followed the reporting guideline for case series.

## Results

During the observation period, 236 ambulatory care patients died by suicide, including 114 (48%) who died by firearm (98 men [86%] and 16 women [14%]; 3 Asian [3%], 3 Black [3%], 1 Hawaiian or Pacific Islander [1%], 3 Hispanic or Latino/a/x [3%], 97 White [85%], and 7 of unknown race or ethnicity [6%]; 57% younger than 65 years), of whom 104 (91%) had utilized care in 1 or more clinics using the MH questionnaire with the firearm question (93 for primary care [82%], 30 for MH specialty care [26%], and 41 for urgent care [36%]) (Table). Sixty-seven firearm suicide decedents (59%) had MH or SUD diagnoses (the target patient-population for the MH questionnaire), 41 (36%) received the firearm question, 38 (33%) answered, and 17 (15%) reported access (Figure). Eighty-four of 122 other suicide decedents (69%) had MH or SUD diagnoses, 51 (42%) received the firearm question, 44 (36%) answered, and 2 (2%) reported access.

**Table.** Sociodemographic Characteristics, Health Care Use, and Prior-Year Diagnoses of 236 Individuals Who Received Ambulatory Care Within the 12 Months Before Suicide Death

Characteristic	No. (%)	
	Firearm suicide (n = 114)	Other means (n = 122)
Age, mean (SD) y	57.4 (1.9)	51.5 (1.6)
Age category, y		
18-39	25 (22)	34 (28)
40-64	40 (35)	60 (49)
≥65	49 (43)	28 (23)
Sex		
Female	16 (14)	52 (43)
Male	98 (86)	70 (57)
Race and ethnicity		
American Indian or Alaska Native	0	1 (1)
Asian	3 (3)	9 (7)
Black	3 (3)	4 (3)
Hawaiian or Pacific Islander	1 (1)	2 (2)
Hispanic, Latino/a/x	3 (3)	5 (4)
White	97 (85)	88 (72)
Unknown	7 (6)	13 (11)
Rural or urban^a^		
Urban	38 (33)	52 (43)
Large suburban	27 (24)	37 (30)
Small suburban or rural	49 (43)	33 (27)
**Any primary care**		
Prior 3 mo	45 (39)	44 (36)
Prior 12 mo	93 (82)	91 (75)
**Any MH specialty care (outpatient)**		
Prior 3 mo	17 (15)	31 (25)
Prior 12 mo	30 (26)	46 (38)
**Any urgent care**		
Prior 3 mo	13 (11)	23 (19)
Prior 12 mo	41 (36)	43 (35)
Diagnoses in prior year^b^		
Disorders		
Depression	49 (43)	70 (57)
Anxiety	46 (40)	63 (52)
Serious mental illnesses	13 (11)	13 (11)
Substance use disorder	31 (27)	25 (20)
Suicide attempt	5 (4)	11 (9)

^a^ Based on a condensed version of the 2013 National Center for Health Statistics county urban-rural categorization.

^b^ The diagnostic codes used to create the analytic data set used for this analysis are publicly available from the Mental Health Research Network (https://github.com/MHResearchNetwork/Diagnosis-Codes). Serious mental illness diagnoses include bipolar, schizophrenia, other psychosis or personality disorders.

**Figure.**
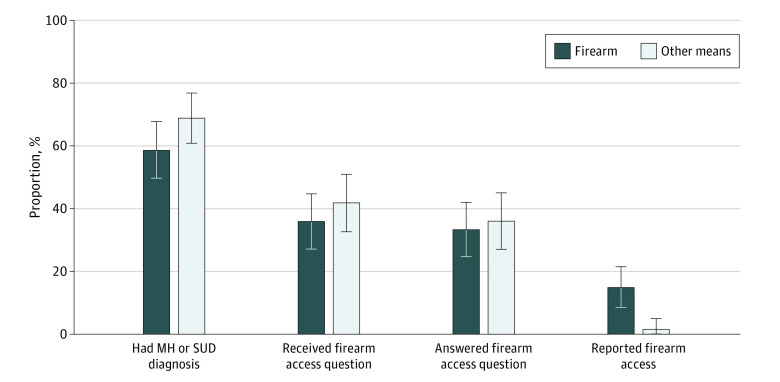
Firearm Access Assessment Outcomes Cumulative proportions of individuals who died by suicide who (1) had a mental health (MH) or substance use disorder (SUD) diagnosis, (2) received the firearm access question (ie, had an electronic health record–documented MH monitoring questionnaire), (3) answered the firearm access question, and (4) reported firearm access.

## Discussion

Our findings have important implications for health care systems that are considering firearm access screening to support suicide prevention. First, this study underscored the potential reach of standardized firearm access questions in primary care clinics, which implemented routine use of this question during the study and where the highest proportion of firearm suicide decedents were seen prior to death. Second, the decision to only ask primary care patients with a MH or SUD diagnosis about firearm access likely resulted in missed opportunities, as many firearm suicide decedents did not have these diagnoses.

This study also highlighted the need to improve how health care systems ask standardized questions. Most patients who died by firearm suicide answered the firearm access question when they received it, confirming our prior finding that patients will answer this standardized firearm access question.^5^ However, more than half of those who answered the question and who subsequently died by firearm suicide reported no firearm access. A study limitation is that we cannot determine whether these individuals acquired access after answering the question or answered no despite having access. Nevertheless, prior qualitative findings suggest that transparency about how firearm access information will be used and building clinician competency and clinician–patient trust may encourage honest reporting, open dialogue, and improved patient-centeredness of this practice.^6^ Assessing patients’ plans to acquire firearms may also be useful. Future work is needed to test language and strategies designed to encourage patient-reported firearm access.

## Notes

Supplement.
eMethods.

eReferences.
