# Supplementary material for: Patient-Reported Firearm Access Prior to Suicide Death
Source: JAMA Netw Open. 2022 Jan 10;5(1):e2142204. doi: 10.1001/jamanetworkopen.2021.42204 (PMC8749466; doi:10.1001/jamanetworkopen.2021.42204)
Supplement: Supplement. — eMethods. eReferences. [file jamanetwopen-e2142204-s001.pdf]

Supplemental Online Content

Richards JE, Boggs JM, Rowhani-Rahbar A, et al. Patient-reported firearm access prior to suicide death. *JAMA Netw Open.* 2022;5(1):e2142204. doi:10.1001/jamanetworkopen.2021.42204

eMethods.

eReferences.

This supplemental material has been provided by the authors to give readers additional information about their work.

© 2022 Richards JE et al. *JAMA Network Open.*

eMethods

Study Design

This case series study examined completion of and responses to a mental health screening question about firearm access among patients who died by suicide. This analysis sought to identify opportunities to improve the clinical process of asking a standard firearm question on a mental health questionnaire to support suicide prevention (Figure). This study design is recommended for examining novel clinical practices (standardized firearm access questions) and their impact on rare outcomes (suicide).[1]

Data Source

The Kaiser Permanente Washington Virtual Data Warehouse (VDW) consists of electronic health medical record (EHR) and insurance claim data for enrolled members. Data on encounters, pharmacy fills, diagnoses, medical tests, and demographics are organized using standard definitions across the [Health care Systems Research Network] and are quality checked locally.[2] These data are linked to official Washington State mortality records using Social Security Numbers or patient names, birthdates, and demographic profiles.

Sample

Adult KPWA patients (patients 18+), who died by firearm suicide between 2016-2019, following implementation of the firearm access question (previously described),[3] who had at least one visit within the KPWA healthcare system in the year prior to death.

Figure: Process outcomes (white boxes) and patient outcomes (shaded boxes), among individuals who received ambulatory care within 12 months prior to suicide death.

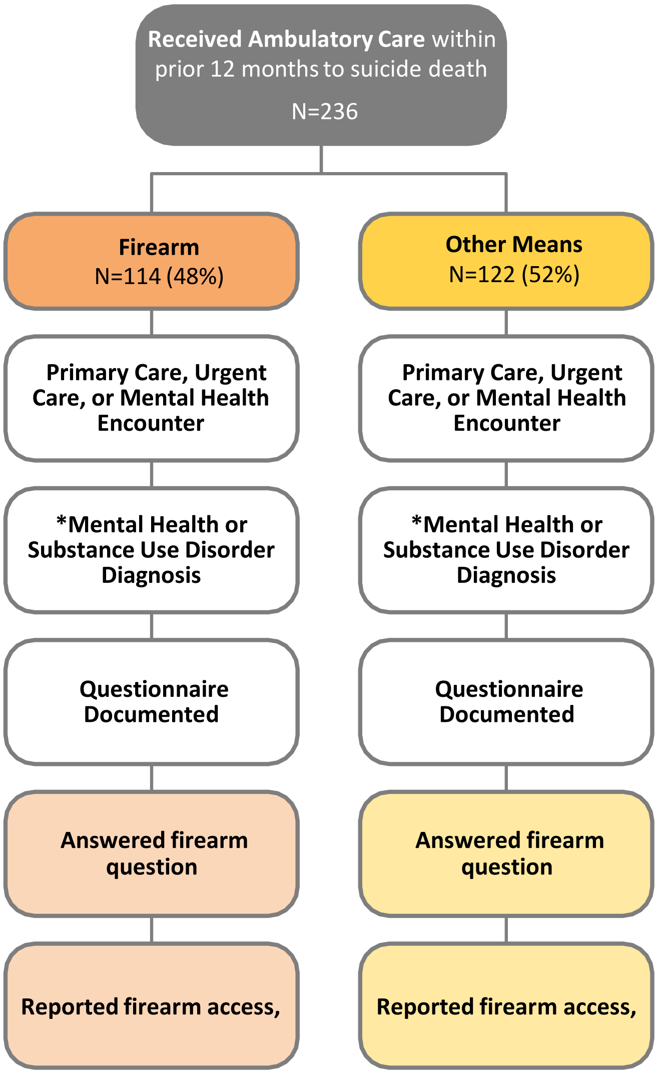

**Target population for receiving the mental health monitoring questionnaire with the firearm access question.*

Analytic Dataset Specifications

Below is a description of the general approach used to assemble the dataset for this descriptive case series analysis.

Step 1: Identify all patients who died by suicide between 1/2016-12/31/2019. Exclude patients <18 at the time of death. Create a binary firearm suicide cause-of-death indicator for firearm suicide cause-of-death, using ICD-10 codes for intentional self-harm by firearm (x72-x74).[4]

Step 2: Link person-level demographic characteristics and prior year diagnoses (previously described)[3] at the time of death, using VDW data and diagnostic code categories previously defined for the Mental Health Research Network. Details available here: https://github.com/MHResearchNetwork/Diagnosis-Codes.

Step 3: Add person-level visit indicators and counts in year prior to suicide death. Create visit count variables for any internal in primary care [PC], outpatient mental health specialty [MH], urgent care [UC] or other specialty settings [OT] in the prior 3, 6, or 12 months (categories are not mutually exclusive). Create indicators for any care in the same settings in the prior 3, 6, or 12 months to suicide death.

© 2022 Richards JE et al. *JAMA Network Open.*

Step 4: Create person-level indicators for 1) MH monitoring questionnaire receipt, 2) firearm question response, and 3) reported firearm access in the 365 days prior to suicide death.

Definition of receipt and response to firearm access question: As previously described,[3] no discrete data element existed in the EHR that could be used to define when the mental health monitoring questionnaire with the firearm access question was administered to patients. Therefore, based on preliminary analyses and consultation with clinical leaders, the presence of a response to one of the two questions screening questions for general anxiety disorder [GAD-2],[5] on the mental health monitoring questionnaire was used to define when patients "received" the mental health monitoring questionnaire. The GAD-2 was used for this purpose because it is specific to the monitoring questionnaire, unlike other questions on this questionnaire which are also included on other frequently used questionnaires. The limitation of this approach is that it misses patients who received the questionnaire and did not complete the GAD-2. Based on consultation with KPWA clinicians and staff, we believe it is uncommon for patients to choose not to answer any questions on the mental health monitoring questionnaire and patients are more likely to answer the GAD-2 (based on clinical experience) than substance use questions (these are more often left blank). Similarly, the presence of a response to one of the two GAD-2 questions without a matching response on the firearm question was used to define non-response to the firearm question.

Institutional Review Board Approval

The Kaiser Permanente Institutional Review Board approved this study and waived the need for patient informed consent and Health Insurance Portability and Accountability Act authorization for access, use, and collection of protected health information from medical records to conduct this study, because use of the protected health information involved no more than a minimal risk to the privacy of individuals.

eReferences

1. Kempen JH. Appropriate use and reporting of uncontrolled case series in the medical literature. *Am J Ophthalmol*. Jan 2011;151(1):7-10 e1. doi:10.1016/j.ajo.2010.08.047S0002-9394(10)00690-2 [pii]

2. The Health Care Systems Research Network. Data Resources. Kaiser Permanente Division of Research (Northern California). Accessed August 19, 2021. http://www.hcsrn.org/en/About/Data/

3. Richards JE, Kuo E, Stewart C, et al. Self-reported Access to Firearms Among Patients Receiving Care for Mental Health and Substance Use. *JAMA Health Forum*. 2021;2(8):e211973-e211973. doi:10.1001/jamahealthforum.2021.1973

4. CMS.gov Centers for Medicare & Medicaid Services. ICD-10-CM, ICD-10-PCS, CPT, and HCPCS Code Sets. Accessed June 24, 2020. https://www.cms.gov/Outreach-and-Education/Medicare-Learning-Network-MLN/MLNProducts/MLN-Publications-Items/ICN900943.html

5. Staples LG, Dear BF, Gandy M, et al. Psychometric properties and clinical utility of brief measures of depression, anxiety, and general distress: The PHQ-2, GAD-2, and K-6. *Gen Hosp Psychiatry*. Jan - Feb 2019;56:13-18. doi:S0163-8343(18)30371-2 [pii] 10.1016/j.genhosppsych.2018.11.003

© 2022 Richards JE et al. *JAMA Network Open.*
